# Effects of Bumetanide on Neurocognitive Functioning in Children with Autism Spectrum Disorder: Secondary Analysis of a Randomized Placebo-Controlled Trial

**DOI:** 10.1007/s10803-022-05841-3

**Published:** 2023-01-10

**Authors:** Dorinde M van Andel, Jan J Sprengers, Marsh Königs, Maretha V de Jonge, Hilgo Bruining

**Affiliations:** 1 Department of Psychiatry, UMC Utrecht Brain Centre, University Medical Centre Utrecht, Utrecht, The Netherlands; 2 Department of Paediatrics, Emma Neuroscience Group, Amsterdam UMC Emma Children’s Hospital, Amsterdam, The Netherlands; 3 Department Education and Child Studies, Faculty of Social and Behavioral Sciences, Leiden University, Leiden, The Netherlands; 4 Child and Adolescent Psychiatry and Psychosocial Care, Emma Children’s Hospital, Vrije Universiteit Amsterdam, Amsterdam UMC, Amsterdam, Netherlands; 5 N=You Neurodevelopmental Precision Center, Amsterdam Neuroscience, Amsterdam Reproduction and Development, Amsterdam UMC, Amsterdam, Netherlands; 6 Levvel, Center for Child and Adolescent Psychiatry, Amsterdam, Netherlands; 7 Department of Child and Adolescent Psychiatry, Amsterdam UMC, University of Amsterdam, Meibergdreef 9, 1105 AZ Amsterdam, the Netherlands

**Keywords:** ASD, Bumetanide, RCT, Child, Cognition, Neurocognitive functioning

## Abstract

We present the secondary-analysis of neurocognitive tests in the ‘Bumetanide in Autism Medication and Biomarker’ (BAMBI;EUDRA-CT-2014-001560-35) study, a randomized double-blind placebo-controlled (1:1) trial testing 3-months bumetanide treatment (≤ 1 mg twice-daily) in unmedicated children 7–15 years with ASD. Children with IQ ≥ 70 were analyzed for baseline deficits and treatment-effects on the intention-to-treat-population with generalized-linear-models, principal component analysis and network analysis. Ninety-two children were allocated to treatment and 83 eligible for analyses. Heterogeneous neurocognitive impairments were found that were unaffected by bumetanide treatment. Network analysis showed higher modularity after treatment (mean difference:-0.165, 95%CI:-0.317 to − 0.013,p = .034) and changes in the relative importance of response inhibition in the neurocognitive network (mean difference:-0.037, 95%CI:-0.073 to − 0.001,p = .042). This study offers perspectives to include neurocognitive tests in ASD trials.

Autism spectrum disorder (ASD) is classified on the basis of persistent dysfunction in social communication and restricted, repetitive behavior. Consistent with this definition, ASD trials generally focus on behavioral outcomes as endpoint measures of efficacy. Yet, neurocognitive dysfunctions have systematically and repeatedly been reported in ASD, but neurocognitive outcome measures are very rarely included in trial designs. Both impairments in social and non-social neurocognitive functioning have been found in children and adults with ASD, suggesting a pervasive pattern of dysfunction in neurocognitive functioning (Demetriou et al., 2018; Velikonja, Fett, & Velthorst, 2019). Impairments in executive functioning (Demetriou et al., 2018; Lai et al., 2017) and working memory (Wang et al., 2017) are among the most prominent and consistent findings, which led to the development of the executive dysfunction hypothesis (Demetriou, DeMayo, & Guastella, 2019; Hill, 2004; Pennington & Ozonoff, 1996). This hypothesis suggests that neurocognitive deficits underlie ASD core symptomatology (Leung, Vogan, Powell, Anagnostou, & Taylor, 2016; E. Pellicano, 2007). Although no single neurocognitive factor can explain the broad range of behavior as disease manifestations in ASD are too complex and fractionated to be explained by a single cause (Happe, Ronald, & Plomin, 2006), research showed that dysfunctions in executive function are important factors for targeted assessments and interventions (Demetriou et al., 2019). This is supported by evidence that neurocognitive functioning is predictive of adaptive functioning (i.e. how well one can perform daily activities and meet every day environmental demands), school attainment and professional career opportunities (Gillespie-Lynch et al., 2012; Gilotty, Kenworthy, Sirian, Black, & Wagner, 2002; Kenny, Cribb, & Pellicano, 2019).

Many neurocognitive tasks tap into overlapping constructs, thereby complicating the detection of more specific neurocognitive effects. Furthermore, neurocognitive assessments are typically analyzed using a univariate approach (i.e. the measurement and analysis of isolated neurocognitive functions), while neurocognitive functions are known to have complex inter-dependency (for a review, see (Meehan & Bressler, 2012)). The conventional univariate approach to neurocognitive assessment therefore provides an oversimplified view of neurocognitive functioning, disregarding the complex interplay in a network of neurocognitive functions, with the risk of missing treatment (side) effects. These observations highlight the challenges and opportunities for neurocognitive assessment in trial designs. In the context of ASD trials, additional challenges are posited by extensive heterogeneity in neurocognitive profiles between and within children with ASD (Elizabeth Pellicano, 2010). As a consequence, neurocognitive treatment effects in ASD are expected to be complex and not straightforward to detect at the group level. This directly emphasizes the importance of more advanced approaches to neurocognitive assessment, which may allow for inter-individual differences in the neurocognitive profile and have the potential to detect more subtle, yet clinically important effects on neurocognitive functioning.

Several new treatment options have emerged that may improve neurocognitive functioning in ASD. These candidates have been derived from animal model studies and are regarded as mechanism-based treatments. One example of such a treatment candidate is bumetanide, a diuretic that is being repurposed for ASD treatment. In several animal models of ASD, bumetanide has shown to reinstate GABAergic inhibition and enhance neuronal oscillations through correction of neuronal chloride homeostasis (Schulte, Wierenga, & Bruining, 2018; Tyzio et al., 2014). Following these observations, a number of randomized controlled trials (RCTs) in children with ASD have shown improvement in ASD traits (Du et al., 2015; Lemonnier et al., 2012; Lemonnier et al., 2017; Sprengers et al., 2021; Zhang et al., 2020). In these RCTs, the effect of bumetanide on neurocognitive functioning was not investigated. An open label study with TSC patients included neurocognitive tests, but found no treatment effects (van Andel et al., 2020). A pilot study using fMRI and eye-tracking in children with ASD showed that 10 months of bumetanide treatment resulted in improved social neurocognitive function (i.e. emotional face processing) (Hadjikhani et al., 2018; Hadjikhani et al., 2015). In patients with drug-resistant epilepsy, 6 months of bumetanide treatment improved performance on spatial memory tests (Gharaylou et al., 2019). In animal studies, bumetanide showed improvements in memory functioning in a valproate induced rat model (Liu et al., 2016) and in Down syndrome (Deidda et al., 2015) and Huntington’s disease mouse models (Dargaei et al., 2018). These disease models have been linked to disturbances in chloride regulation and/or GABA polarity (Cepeda et al., 2019; Patel, Lukkes, & Shekhar, 2018; Sohal & Rubenstein, 2019; Souchet et al., 2015) and therefore suggest that bumetanide may restore certain aspects of neurocognitive functioning.

The aim of this exploratory study was to test the effect of bumetanide treatment on neurocognitive functioning in children with ASD. This study is performed as secondary analyses of the ‘Bumetanide in Autism Medication and Biomarker’ (BAMBI) study in which the effects of 3 months of bumetanide treatment on behaviour and electroencephalography were tested as well. We included a broad test battery to cover a range of important neurocognitive domains. As children with ASD show highly variable configuration of neurocognitive profiles, we expect complex multifactorial effects of bumetanide on cognitive functioning, and neurocognitive functions to show complex interplay. We therefore undertook an analysis strategy utilizing principal component analysis to cluster neurocognitive domains and additionally applying network analysis to capture treatment effects on the organization of neurocognitive functions in a network.

## Methods

This exploratory study is part of the BAMBI study, of which the primary behavioral outcome has been previously reported (Sprengers et al., 2021). The BAMBI trial was a mono-center, parallel-group, patient-randomized, double-blind, placebo-controlled phase-2 superiority trial testing the effect of bumetanide treatment during 91 days, followed by 28-day wash-out. Detailed information on the study design, sample selection procedures and sample characteristics can be found in Sprengers et al. (2021). The trial was conducted at the University Medical Centre Utrecht (UMCU), the Netherlands, a nation-wide tertiary out-patient center, approved by the medical ethical committee of the UMCU, and conducted in accordance with the provisions of the declaration of Helsinki and Good Clinical Practice. All participants or their legal representatives signed informed consent.

### Participants

In brief, inclusion criteria were children aged 7–15 years with an expert confirmed ASD diagnosis according to DSM-IV-TR (i.e. autism, Asperger syndrome or pervasive developmental disorder – not specified otherwise [PDD-NOS]) or DSM-5 criteria and either an Autism Diagnostic Observation Scale-2 (ADOS-2) module 3 algorithm total score ≥ 6, Social Responsiveness Scale-2 (SRS-2) T-score ≥ 60 or a confirmed diagnosis by an independent in-house child-psychiatrist. Exclusion criteria are mentioned in (Sprengers et al., 2021). Most important exclusion criteria were an IQ < 55 (and IQ < 70 for neurocognitive analyses in this paper because of validity of the test battery); psychoactive medication use less than eight weeks prior to screening visit (except chronic melatonin treatment); start of any new therapy for developmental disorder problems. Furthermore, children were allowed to receive care as usual.

### Study Design

Eligible participants were allocated to receive bumetanide or placebo treatment. Patients, parents, healthcare providers and outcome assessors were masked for randomization. Participants received bumetanide liquid formulation (0.5 mg/ml) or placebo formulation matched for taste, smell and viscosity, albeit without diuretic properties. The formulation was twice-daily administered orally with minimally 6 h between the doses. Children < 30 kg started with twice-daily 0.015 mg/kg bumetanide or an equivalent volume of the placebo formulation. Children ≥ 30 kg received twice-daily 0.5 mg bumetanide or placebo (i.e. 1ml). When blood analysis showed no abnormalities at D7, the dosage was doubled. All participating children were supplemented with 0.5mmol/kg potassium chloride < 30 kg, or twice-daily 8mmol potassium chloride ≥ 30 kg.

During the first visit, baseline clinical outcomes were assessed (e.g. questionnaire) and during subsequent baseline assessment, neurocognitive measurements were performed. Participants returned for outcome evaluations at the end of the 91-day medication phase (with neurocognitive assessment) and at the end of the 28-day wash-out period (without neurocognitive assessment).

Study safety was overseen twice a year by the Data Safety Monitoring Board (DSMB) of the UMC Utrecht. This study was registered with the EudraCT trial registry (2014-001560-35).

### Outcomes

#### Neurocognitive Measures

An abbreviated Wechsler Intelligence Scale for Children-III (WISC-III) intelligence test was conducted to screen intelligence for study exclusion (when no IQ test was performed in the previous two years). Since we could not formulate an a priori hypothesis on the nature and extent of neurocognitive effects of bumetanide in ASD, we composed a test battery measuring a broad range of neurocognitive functions. The following domains were selected: information processing speed and attention, memory and executive functioning using a balanced battery assessing both auditory and visual processing. The neurocognitive tasks, the corresponding functions they intend to measure and variables used in the analyses are depicted in Supplementary Table 1. The duration of the complete neurocognitive battery was approximately 120 min, which was performed in the morning in a quiet room with one trained psychological assistant supervised by a clinical psychologist. Basic processing speed, response inhibition and attentional flexibility were measured with subtasks of the Amsterdam Neuropsychological Task battery (ANT). Working memory for auditory and visual information were assessed with respectively the digit span of the WISC-III (WISC-III-DS) and spatial span of the Wechsler Nonverbal Scale of Ability (WNV-SS). Verbal and visual learning and memory were examined with the Rey Auditory Verbal Learning Test (RAVLT) and the Rey Visual Design Learning Test (RVDLT). The ‘post-response interval’ between the different trials of the ANT tasks was event-driven. Different versions of the RAVLT were used to minimize practice effects.

#### Behavioral Questionnaires

In addition to the neurocognitive task battery, a behavioral equivalent of neurocognitive processes was measured. The Behavior Rating Inventory of Executive Functioning (BRIEF; both parent and teacher reported) was included to measure executive functioning behaviors in school and home environments. Core trait behavior was measured with the Social Responsiveness Scale 2 (SRS-2) and the Repetitive Behaviors Scale – Revised (RBS-R).

### Statistical Analysis

These secondary analyses were part of the statistical plan of the BAMBI study, except for the network analysis. First, we defined neurocognitive dysfunction at baseline (i.e. z-scores ≤-1) including only the tasks for which norm-data (based on a neurotypical comparison group as presented in the test-manuals) were available (i.e. no norm-data were available for computed, difference or domain scores). It is important to note that norm-data were predominantly unavailable for participants aged > 12 years, which might cause bias. Significance was tested with one sample chi-square tests assuming 16% deviation in the norm population (i.e. z-score ≤-1). Further analyses of treatment effect included raw data in order to include all participants and all tasks. Second, contrast scores were computed to isolate specific neurocognitive functions (see Supplementary Information 1). Third, domain scores were extracted using data-driven principal component analysis (PCA) with varimax rotation from the Psych package in R. The number of components (i.e. neurocognitive domains) to extract was determined using the elbow method and the cumulative variance explained from the eigenvalues histogram (*R*^2^ ~ 70%). Subsequently, each component was labeled as a neurocognitive domain based on the set of test scores that made the strongest contribution to the components, as measured in terms of η^2^. The boundary for this set of test scores was set at the largest drop in η^2^ between two subsequent variables in the scree plot. The eight neurocognitive domains resulting from this procedure were used in subsequent analyses (see the Results section). Fourth, we tested treatment effect on the neurocognitive domains using generalized linear models (GLMs), whereas the BRIEF-questionnaires also included wash-out data and were analyzed with linear mixed models (LMMs). The models included baseline measurement, age and sex to correct for potential confounding factors. Assumptions were tested by residual plots. Estimated means per treatment group and mean differences after treatment were calculated with 95% confidence intervals and p-values. Treatment interactions with sex and age were tested with likelihood ratio tests. No correction for multiple testing was applied, due to the exploratory nature of the analyses, accepting a higher risk for finding false-positive results. The results are regarded as hypothesis generating for more dedicated trial outcome designs. Finally, we explored treatment effects on neurocognitive network organization using neurocognitive network analysis (for detailed description of the methodology of the network analysis see (Konigs, Verhoog, & Oosterlaan, 2021)). This is an innovative approach that uses network analysis on an individual’s neurocognitive data from a single assessment (see textbox 1 for an explanation of terminology in graph theory). Nodes represent neurocognitive variables, edges represent the connectivity between neurocognitive variables (i.e. the inverse of the absolute difference in normalized scores of the neurocognitive variables). All scores are normalized compared to the study population. Directionality of scores is matched where positive scores mean improvement. A connectivity matrix is constructed by the intra-individual difference in relevant test scores. The matrix is corrected for chance connectivity by use of a neurocognitive skeleton which was formed by population level associations between neurocognitive variables. Thresholding was applied and the network was closed with the use of minimal spanning trees. Global network parameters (strength, modularity, assortativity, characteristic path length, transitivity, and smallworldness; reflecting organization of neurocognitive functions in the network as a whole) and local network parameters (hubness score, reflecting the relative importance of each neurocognitive function in the network) were calculated. To limit the number of comparisons, we selected the analysis regarding local network parameters to the 20% most influential neurocognitive functions in the network. We tested global and local network parameters for treatment effects with the same GLMs as described for the analysis of neurocognitive domains. All GLMs analyses were performed with SPSS v25 (IBM, Corp., Armonk, NY), LMMs with SAS v9.4 (SAS Institute, Cary, NC) and PCA and network analyses were performed with the ‘igraph’ and ‘qgraph’ packages in R v3.6.


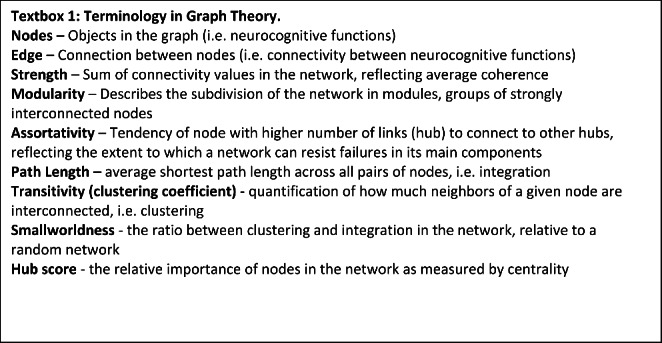


## Results

### Participant Characteristics

**Fig. 1:**
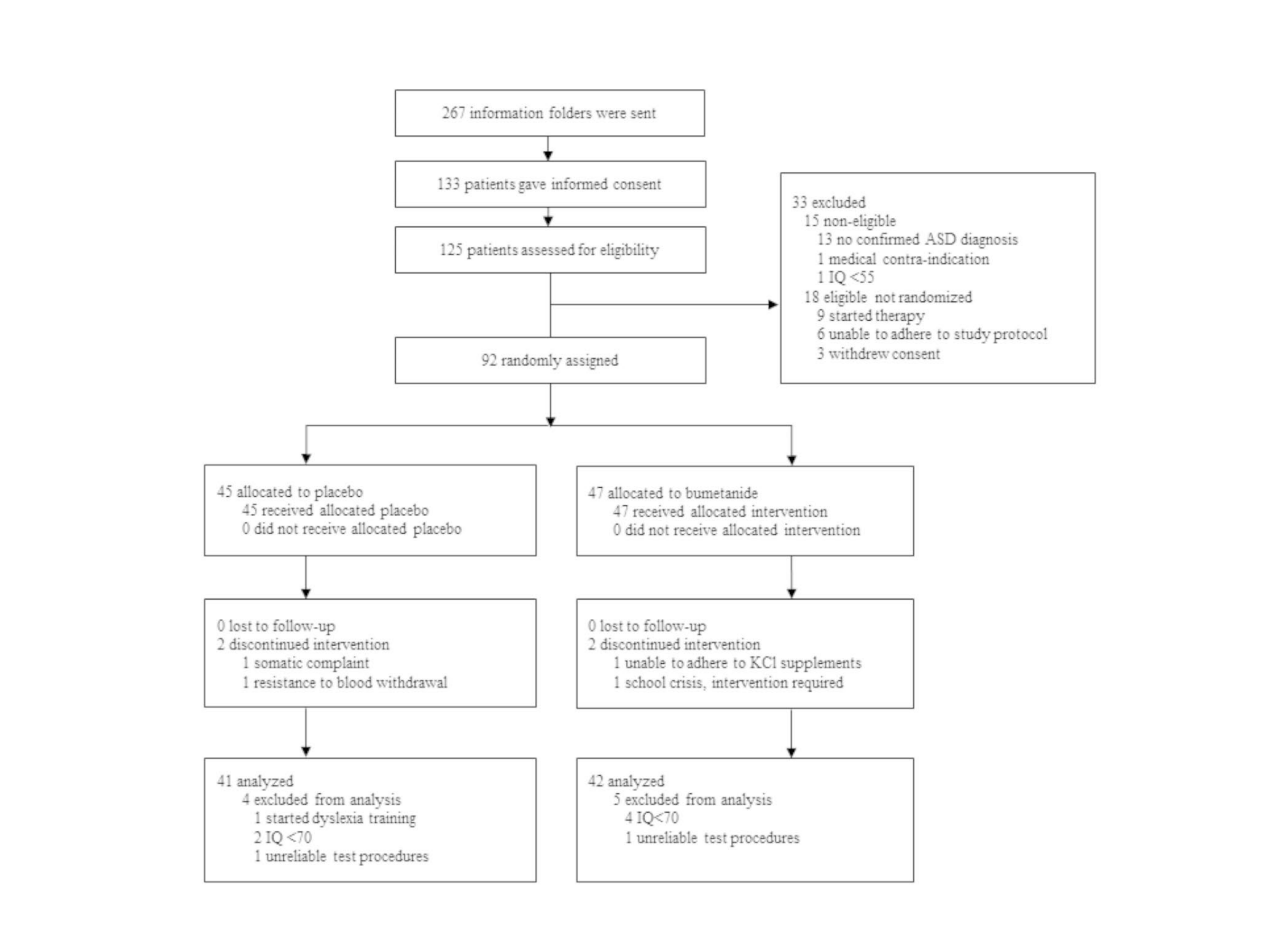
CONSORT Flow Diagram

Participants were enrolled between June 21st 2016 and December 6th 2018 and 92 participants were randomly allocated to treatment. Four participants discontinued the study intervention, but were included in the ITT-analysis. Six participants were excluded for neurocognitive analyses because their IQ was below 70 and 3 due to unreliable measurements (see Fig. 1). The resulting sample that is analyzed in this paper is depicted in Table 1.

**Table 1:** Baseline characteristics of the BAMBI population

	Placebo group *(n* = 41)	Bumetanide group *(n* = 42)	Total *(n* = 83)
Age (y, SD)	10.5 (2.4)	10.5 (2.4)	10.5 (2.4)
Male (%)	29 (70.7)	32 (76.2)	61 (73.5)
Female (%)	12 (29.3)	10 (23.8)	22 (26.5)
FSIQ (SD)	104.9 (18.4)	103.3 (17.7)	104.1 (18.0)
SRS-2 (SD)	89.4 (18.8)	88.7 (21.0)	89.0 (19.8)
ADHD (%)	7 (17.1)	10 (23.8)	17 (20.5)

*Note.* Data are mean (SD) or N (%). Y = year; ADHD = Attention-Deficit/Hyperactivity Disorder; FSIQ = full scale intelligence quotient; SRS-2 = Social Responsiveness Scale (range 0-195; higher score is more affected).

### Outcomes

**Fig. 2:**
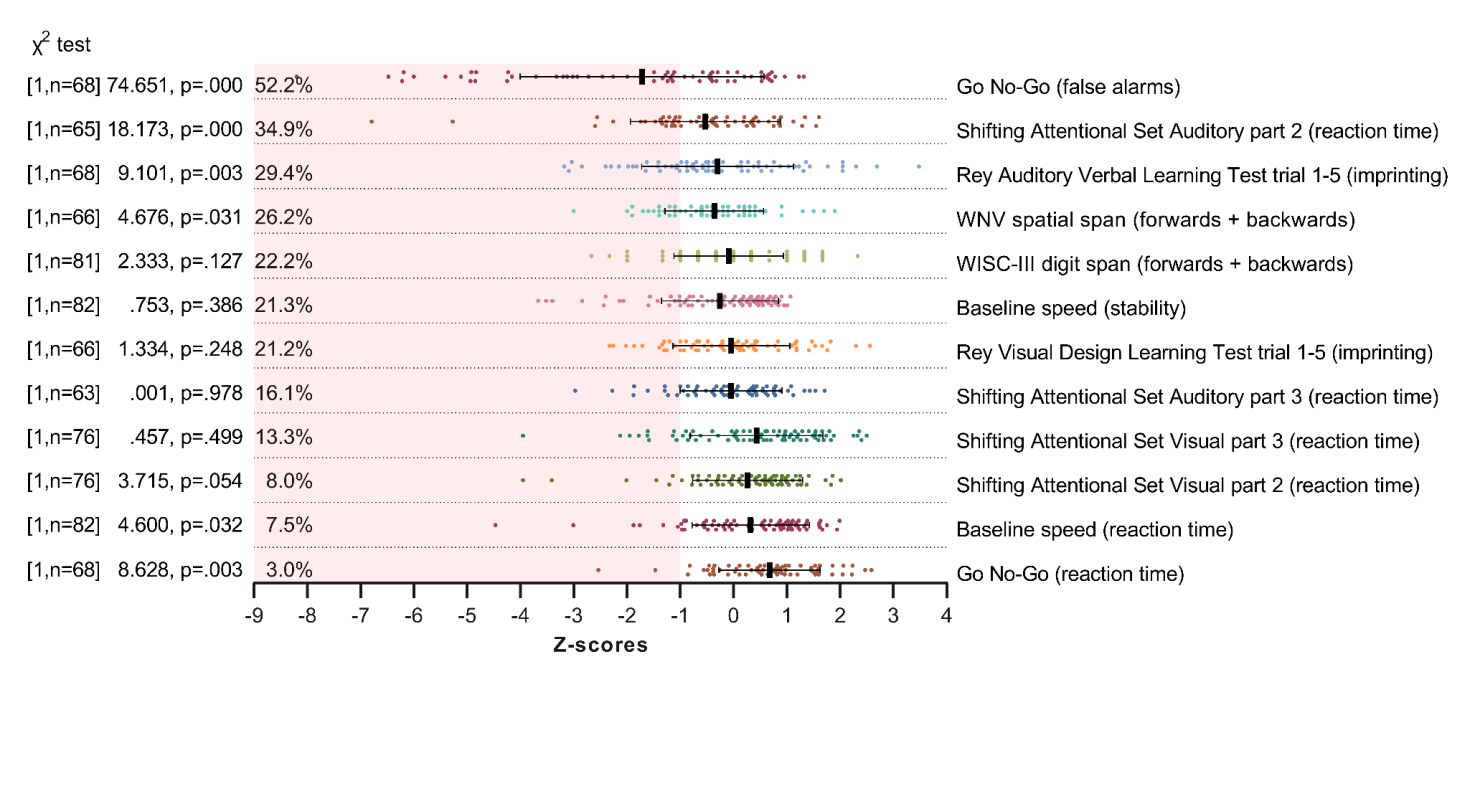
Percentage of deviant Z-scores in neurocognitive performance at baseline compared to neurotypical children. (*Notes*. Baseline score of neurocognitive tasks (non-computed scores) ≤-1 standard deviation are depicted in the shaded area. Domains are arranged in order from highest to lowest percentage of deviant z-scores. Black vertical bars are means, black whiskers represent standard deviation. Significance is tested with one sample chi-square tests assuming 16% deviation in the norm population.)

Figure 2 presents an overview of neurocognitive dysfunction at baseline (i.e. z-scores ≤-1 based on available norm-data) arranged in order from highest to lowest percentage deviance in z-scores. The most prevalent dysfunction was observed in the number of false alarms (in 52.2% of the participants) on the GNG task, indicative of dysfunction in response inhibition (χ^2^ [1, n = 68] = 74.65, p = .000), followed by dysfunction on SSA task 2 measuring auditory prepotent response inhibition (34.9%, χ^2^ [1, n = 65] = 18.17, p = .000) and RAVLT measuring verbal recall (number of items: 29.4%, χ^2^ [1, n = 68] = 9.10, p = .003). The least frequently observed dysfunctions were observed in reaction time on the GNG task (3.0%, χ^2^ [1, n = 68] = 8.63, p = .003) and the baseline speed task (7.5%, χ^2^ [1, n = 82] = 4.6, p = .032). These baseline results confirm extensive variability in neurocognitive profiles among participants and dysfunction in (prepotent) response inhibition.

The PCA yielded eight components that together explained 76% of the variance of all neurocognitive tasks. We named these components according to the common neurocognitive domains they represented: information processing and control, memory imprinting, visual memory, verbal memory, visual working memory, verbal working memory, attentional flexibility motor inhibition. Supplementary Table 2 shows the concomitant factor loadings.

Analyses of treatment effect after 3 months treatment with generalized linear models did not show a superior effect of bumetanide versus placebo on any of the neurocognitive domains (p > .275; see Table 2). Secondary analyses with sex and age did not show interaction effects (p > .07, data not shown).

**Table 2:** Treatment effect after 91 days for the bumetanide and placebo group

	Placebo group		Bumetanide group		Treatment effect	p-value
	Baseline	D91	Baseline	D91		
n	41	41	42	42		
**Domain 1: Information Processing and Control**						
Mean	.03 (.94)	− .13 (1.09)	.13 (1.01)	− .03 (.85)	− .062 (-.325 to 0.201)	.646
**Domain 2: Memory Imprinting**						
Mean	− .01 (1.04)	.22 (.87)	− .26 (.91)	.06 (1.03)	.030 (-0.302 to 0.361)	.861
**Domain 3: Visual Memory**						
Mean	.00 (1.02)	.05 (1.34)	.11 (.84)	.16 (.97)	.248 (-.229 to 0.725)	.308
**Domain 4: Verbal Memory**						
Mean	− .01 (.88)	− .08 (.97)	.02 (1.0)	.07 (1.03)	− .168 (-0.586 to 0.249)	.429
**Domain 5: Visual Working Memory**						
Mean	.01 (1.27)	.02 (.85)	− .06 (.85)	.03 (.64)	− .025 (-0.324 to 0.274)	.868
**Domain 6: Verbal Working Memory**						
Mean	.05 (1.07)	.21 (.85)	− .24 (1.12)	− .02 (.80)	.190 (-0.151 to 0.530)	.275
**Domain 7: Attentional Flexibility**						
Mean	− .37 (1.22)	.09 (.90)	.03 (.85)	.24 (1.01)	.010 (-0.350 to 0.369)	.957
**Domain 8: Motor Inhibition**						
Mean	− .15 (1.03)	.31 (.63)	− .31 (1.20)	.15 (.83)	.103 (-0.161 to 0.367)	.444

*Notes*: Generalized linear models on neurocognitive components after 91 days of bumetanide or placebo treatment. Data are means (SD). Data is shown for the participants intention-to-treat population. Treatment effects are measured with factors: Treatment and Sex; covariates: baseline score and Age; and shown with (95% CI). Negative values favor bumetanide treatment, positive values favor placebo treatment. Significance level is p<.05.

To test whether parent and teacher-reported questionnaires of neurocognitive functioning showed treatment effects, the BRIEF was analyzed. Based on total scores we did not find a difference in symptom severity after bumetanide treatment (Supplementary Table 3). Supplementary Tables 4 and 5 show changes in all subscales after treatment and wash-out.

Next, the complex inter-dependency between neurocognitive functions was investigated using network analysis. The average neurocognitive network is displayed in Supplementary Fig. 1. Analyses of treatment effect on neurocognitive network organization (Table 3) did reveal a significant effect on the global network parameter modularity (standardized mean difference = 0.42, p = .034), while no effects were found for strength, assortativity, characteristic path length, transitivity, and smallworldness. The bumetanide group did show higher modularity after treatment compared to the placebo group, indicating a stronger degree of subdivision of the neurocognitive network into specialized modules. In contrast, no effects were observed in terms of total connectivity in the network (strength), hierarchy (assortativity), integration (characteristic path length), clustering (transitivity) or balance between integration and clustering (smallworldness).

Considering local network parameters, the following neurocognitive functions did show the highest hubness score, indicating that these functions have high relative importance in the neurocognitive network: visual baseline speed (SSV 1 RT), baseline speed (BS RT), stability of baseline speed (BS SD), auditory baseline speed (SSA 1 RT), visual recall (RVDLT imprinting) and response inhibition (GNG biased RT). We did find a treatment effect on response inhibition (GNG biased RT; standardized mean difference = 0.44, p = .042), while no effects were found regarding the other network hubs. Regarding response inhibition, the bumetanide group did show increased hubness after treatment whereas the placebo group did show decreased hubness after treatment. This finding suggests that bumetanide treatment may increase the relative importance of response inhibition in the neurocognitive network.

In the children allocated to bumetanide, both change in modularity and change in response inhibition hubness did not show a correlation with change in ASD behavior measured by the SRS-2 (respectively ρ = 0.239, p = .154; ρ = 0.032, p = .849) or the RBS-R (respectively ρ = 0.114, p = .508; ρ = 0.111, p = .519).

**Table 3:** Treatment effect on neurocognitive network organization after 91 days

	Placebo group		Bumetanide group		Treatment effect	p-value
	Baseline	D91	Baseline	D91		
Global network parameters						
n	41	41	42	42		
Characteristic path length						
Mean	.75 (.02)	.75 (.02)	.75 (.02)	.75 (.02)	.002 (-.006 to .009)	.660
Assortativity						
Mean	.04 (.04)	.04 (.05)	.04 (.04)	.03 (.05)	.008 (-.011 to .027)	.398
Strength						
Mean	24.88 (9.16)	22.60 (9.73)	24.17 (8.1)	21.65 (8.74)	.678 (-3.111 to 4.466)	.726
Transitivity						
Mean	.35 (.01)	.35 (.01)	.35 (.01)	.35 (.01)	− .002 (-.007 to .003)	.376
Modularity						
Mean	1.56 (.40)	1.43 (.30)	1.56 (.39)	1.59 (.43)	− .165 (-.317 to − .013)	.034*
Smallworldness						
Mean	.54 (.02)	.54 (.04)	.53 (.02)	.54 (.02)	− .002 (-.015 to .011)	.752
**Local network parameters**						
n	41	41	42	42		
SSV 1 RT						
Mean	.41 (.10)	.42 (.08)	.42 (.11)	.44 (.09)	− .019 (-.056 to .017)	.299
BS RT						
Mean	.43 (.06)	.41 (.09)	.41 (.09)	.39 (.11)	.016 (-.026 to .059)	.452
BS SD						
Mean	.42 (.06)	.39 (.09)	.39 (.11)	.37 (.11)	.024 (-.017 to .066)	.246
SSA 1 RT						
Mean	.39 (.10)	.39 (.10)	.40 (.10)	.38 (.10)	.000 (-.041 to .041)	.998
RVDLT imprinting						
Mean	.42 (.09)	.38 (.11)	.39 (.11)	.37 (.13)	− .005 (-.051 to .042)	.844
GNG biased RT						
Mean	.37 (.09)	.36 (.10)	.39 (.08)	.40 (.06)	− .037 (-.073 to − .001)	.042*

*Notes*. Generalized linear models on global and local (top 6 hubness) network parameters after 91 days of bumetanide or placebo treatment. Data are means (SD). Data is shown for the intention-to-treat population. Treatment effects are measured with factors: Treatment and Sex; covariates: baseline score and Age; and shown with (95% CI). Significance level is p<.05. Abbreviations. BS: baseline speed; GNG: Go No-Go; RT: reaction time; RVDLT: Rey Visual Design Learning Test; SD: stability; SSA: Shifting Attentional Set Auditory; SSV: Shifting Attentional Set Visual.

## Discussion

Consistent with existing literature, we found that this cohort of children is associated with heterogeneous impairments in neurocognitive functioning. Treatment did not result in physical or mental side effects, other than expected mild reversible diuretic effects. Principle component analysis (PCA) revealed eight neurocognitive domains, on which no bumetanide treatment effects we found. However, effects of treatment on neurocognitive network organization were detected by increased specialization (i.e. higher modularity) and diametrical effects of bumetanide versus placebo on the relative importance of response inhibition in the network.

There is no consensus on cognitive test battery in ASD research and no a-priori effects of bumetanide on neurocognitive functioning were available. Therefore, a comprehensive neurocognitive test battery was used consisting of auditory and visual tasks covering information processing speed and attention, memory and executive functioning (Lai et al., 2017; Velikonja et al., 2019; Wang et al., 2017). The sample showed heterogeneous baseline neurocognitive impairments with significant dysfunction in verbal recall, response inhibition and auditory prepotent response inhibition compared to an age-matched neurotypical comparison group. In contrast, this ASD cohort showed decreased reaction times in the response inhibition task, in which they were faster compared to neurotypical children. However, this concurred with high rates of false alarms, which illustrates the difficulty when analyzing neurocognitive tasks when the outcomes show an intricate trade-off complicating linear analysis.

### Absolute Treatment Effects

We first adhered to common randomized controlled trial analysis methodology by regarding the *absolute effects* on the test outcomes. The PCA showed logical clustering of tasks in eight separate domains in line with the theoretical constructs. The absence of treatment effects on individual neurocognitive tasks may also be due to the limited duration of treatment (i.e. 3 months) for improvements to be established, or the study being underpowered to detect small treatment effect sizes in this study population. Bumetanide treatment did furthermore not deteriorate any neurocognitive outcome, which has been shown for several psychoactive drug treatments (Fung et al., 2016; Moavero, Pisani, Pisani, & Curatolo, 2018). This indicates that bumetanide has no adverse cognitive effects since no slowing of motor responses were observed. Clinical observations of the study participants and anecdotal reports of their parents frequently described them as being “more aware” or “more attentive”, which effects were, when present, not captured by these conventional analyses.

### Relative Treatment Effects

Second, we performed network analysis to analyze whether *relative effects* were evident, i.e. we determined whether a shift in neurocognitive function relationships occurred through treatment. With this novel strategy, we identified significant changes in the global network parameter modularity and the local network parameter (i.e. hubness score) of response inhibition compared to placebo, albeit not corrected for multiple comparisons. As a consequence, there is a risk of presenting false-positive findings consistent with the exploratory design of the study. At this stage, we could not relate the observed network changes to clinical outcome. In ASD research, it has been notoriously difficult to find correlations between neurocognitive test results and behavior scores by questionnaires (Jones et al., 2018). However, previous work has shown that network parameters have relevance for IQ and the presence of behavioral problems in adults (Konigs et al., 2021). Nevertheless, the reported effect on modularity suggests that the neurocognitive network adapts to a more specialized organization in response to bumetanide, while the relative importance of response inhibition decreased. The real-life consequences of such neurocognitive network reorganizations, for instance effects on daily life functioning or school performance, should be investigated in future work.

Although speculative, these subtle neurocognitive changes may fit the suggested mechanism of action of bumetanide on excitatory and inhibitory (E/I) balance in neuronal networks. Studies in computational psychology and electrophysiology provide evidence for tight regulation of the balance between excitation and inhibition in order to maintain efficient neurocognitive information processing (Deneve, Alemi, & Bourdoukan, 2017; Yizhar et al., 2011). Small deviations are speculated to influence neurocognitive functioning (Deneve et al., 2017) and dysregulation of this balance has been implicated in several neurological diseases (Alzheimer, Parkinson, Huntington, epilepsy) and psychiatric conditions (ASD, Down syndrome, schizophrenia) (Cepeda et al., 2019; Patel et al., 2018; Sohal & Rubenstein, 2019; Souchet et al., 2015), all of which are characterized by neurocognitive dysfunction.

To our knowledge this was one of the first ASD trials with children to include neurocognitive measurements in addition to behavioral outcomes (Handen, Johnson, McAuliffe-Bellin, Murray, & Hardan, 2011). During this trial we encountered multiple limitations and recommendations for future studies. First, 3-months of treatment might have been too short to establish profound neurocognitive changes. Parents consistently reported gradual treatment effects in terms such as “being able to catch up developmental processes” or “being more aware”, which may need more time to become detectable in neurocognitive tests. Indeed, other psychoactive drugs, such as antidepressant, antipsychotic and anti-epileptic drugs also show gradual effects taking several months to become apparent. In comparison, studies with stimulants are by definition associated with more immediate neurocognitive effects (Mehta, Goodyer, & Sahakian, 2004). Bumetanide may have a more delayed onset of effects on neurocognitive functioning due to the time course of plasticity alterations in neurocognitive networks with altered GABAergic transmission (Hadjikhani et al., 2018). Second, the variability of neurocognitive profiles at baseline might complicate the detection of effects, which is inherently related to the unselected sample design. Third, the small prevalence of co-occuring ADHD classifications and the absence of outcome measures for ADHD or other relevant co-occuring traits, limits the possibility to evaluate the impact of bumetanide on co-occurring behaviors and hence conclusions on possible mediating constructs. For future studies we advise to implement cross-diagnostic approaches to measure treatment effects on broader neurodevelopmental traits. Fourth, there is a large variation in neurocognitive tasks and scripts, the majority of which was developed to characterize neurocognitive dysfunctions rather than to detect treatment effects. As a consequence, most test are performed under ideal circumstances, minimalizing the chance of false positive findings at the expense of ecological validity. Moreover, longitudinal testing would be preferred at multiple time-points rather than the two in the current design.

In conclusion, this study showed putative changes in neurocognitive network organization after treatment, which may imply subtle neurocognitive effects of bumetanide. Extended treatment duration and further development of the battery and advanced analyses such as network analysis come forward as important considerations for future trial study designs when testing mechanism-based treatments in these heterogeneous neurodevelopmental disorder populations.
